# Thermal injury in endoscopic ear surgery between reality and fiction

**DOI:** 10.1007/s00405-025-09332-w

**Published:** 2025-04-21

**Authors:** Waleed Moneir, Reham El-ekiaby, Mohamed Elkahwagi

**Affiliations:** 1 Mansoura University, Mansoura, Egypt; 2 Damietta University, Damietta, Egypt

**Keywords:** Endoscopic ear surgery, EES, Thermal injury, Tympanoplasty, Hearing

## Abstract

**Objective:**

Endoscopic ear surgery (EES) is engaged nearly in all otology procedures in this era. The widespread application is faced by raised drawbacks that EES can induce thermal injury to the inner ear structures.

**Methods:**

This retrospective study investigates the effect of endoscopic tympanoplasty on the postoperative sensorineural element of hearing and other inner ear functions. Cases of endoscopic tympanoplasty admitted to the tertiary referral center in the period of the study were included. Important audiologic data were collected including the preoperative and postoperative bone conduction threshold and air bone gap. The total endoscopic usage time during surgery was collected. Appropriate statistical testing was performed using SPSS 20.

**Results:**

The study included 51 patients who had endoscopic tympanoplasty. The mean age was (33.65 ± 10.840) years. The study showed no statistically significant difference between the preoperative and postoperative bone conduction threshold. In addition, Pearson correlation test showed no statistical association between the total endoscopic usage time and the postoperative bone conduction threshold. No significant vertigo nor facial nerve affection were observed in the postoperative period.

**Conclusion:**

Endoscopic tympanoplasty as an example of EES does not affect the inner ear structures, clinically described as it does not affect the postoperative sensorineural hearing, facial nerve function nor the balance.

## Intoduction

Endoscopic ear surgery (EES) had a great revolution in the past few years with engagement in most otology operations due to its great benefits. The widespread application of EES is a result of getting the endoscopic advantages of a wide-angled view and the ability to look around the corners in the middle ear [1–4]. Application of EES started in the minor and basic procedures like ventilation tubes insertion and stapedotomy [5]. However, its application had a great revolution during the past few years to be engaged in tympanoplasty, ossiculoplasty, cholesteatoma surgery, facial nerve decompression and vestibular schwannoma surgeries [5, 6]. Several meta-analyses and reviews of EES emphasize the safety of the method being a minimally invasive procedure together with low risk [7, 8]. Avoidance of soft tissue dissection decreases the postoperative morbidity and gives a chance for better healing [9].

A major area of concern in the drawbacks of EES is the generation of heat by the light source attached to the endoscope. This heat can be absorbed by the endoscope, and the heated endoscope together with the light source itself may damage surrounding tissue [10, 11]. Few studies tried to investigate the thermal risk associated with the use of oto-endoscopes [12, 13], such as deterioration of inner ear function and facial palsy [14]. The endoscope with a larger diameter transmits more heat and this can theoretically increase the severity of damage to the inner ear. One study postulated that LED light sources are associated with less significant temperature rises and have a more stable output with increasing power than xenon light sources [15].

Tympanoplasty is a commonly used procedure for the treatment of chronic otitis media, and it involves eradication of the disease in the middle ear, repair of the perforated tympanic membrane, and management of conductive hearing loss resulting from ossicular problems [16]. Postauricular microscopic approach is the commonly used one for tympanoplasty but utilizes much soft tissue dissection to reach the middle ear. The microscopic transcanal approach is limited by the narrowest segment of the canal and the view is usually limited [17]. Transcanal endoscopic approach for tympanoplasty is getting more popularized due to being a more minimally invasive surgery [18]. It provides wide angled view of the spaces in the middle ear, ossicles, and ventilation routes with limited soft tissue dissection [19].

The aim of this study is to explore the effect of heat generated during endoscopic tympanoplasty on the middle and inner ear structures investigating any postoperative sensorineural element hearing loss, facial palsy or significant vertigo.

## Methods

To address the purpose of the study, we conducted this retrospective study on cases that had endoscopic tympanoplasty in Mansoura University Hospitals between January 2023 and February 2024. The institutional review board approved the study with a number of R.24.12.2978. Cases of chronic inactive suppurative otitis media with tympanic membrane perforation who required tympanoplasty were included in the study. Patients with age between 18 and 60 years old and those who had surgery via endoscopic technique only were included. Patients with active infection who needed mastoidectomy in addition were excluded. Patients with incomplete data or those who missed the follow up period were also excluded. The preoperative study variables included age, gender, size and site of the tympanic membrane perforation, bone conduction threshold and the air bone gap. The Postoperative study variables included facial nerve function, bone conduction threshold and air bone gap.

### Surgical technique

**Fig. 1:**
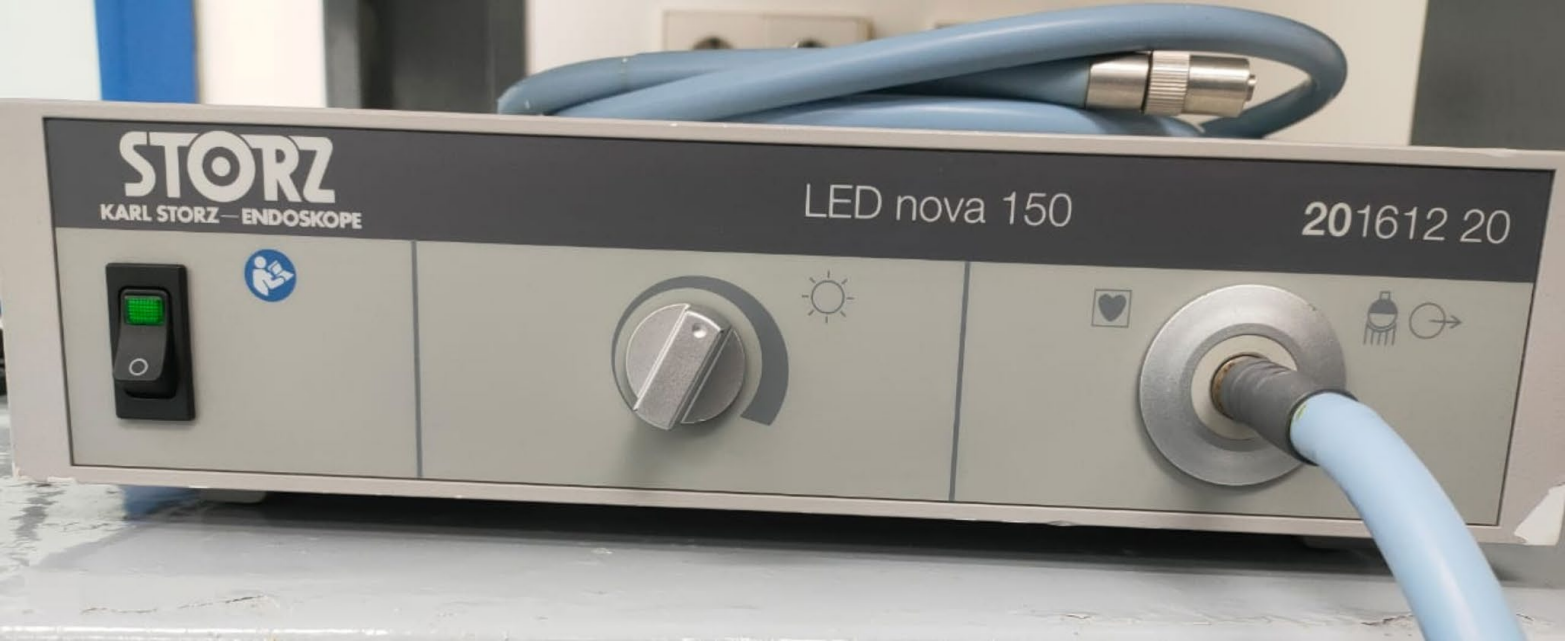
The LED light source used adjusted on 50% illumination

**Fig. 2:**
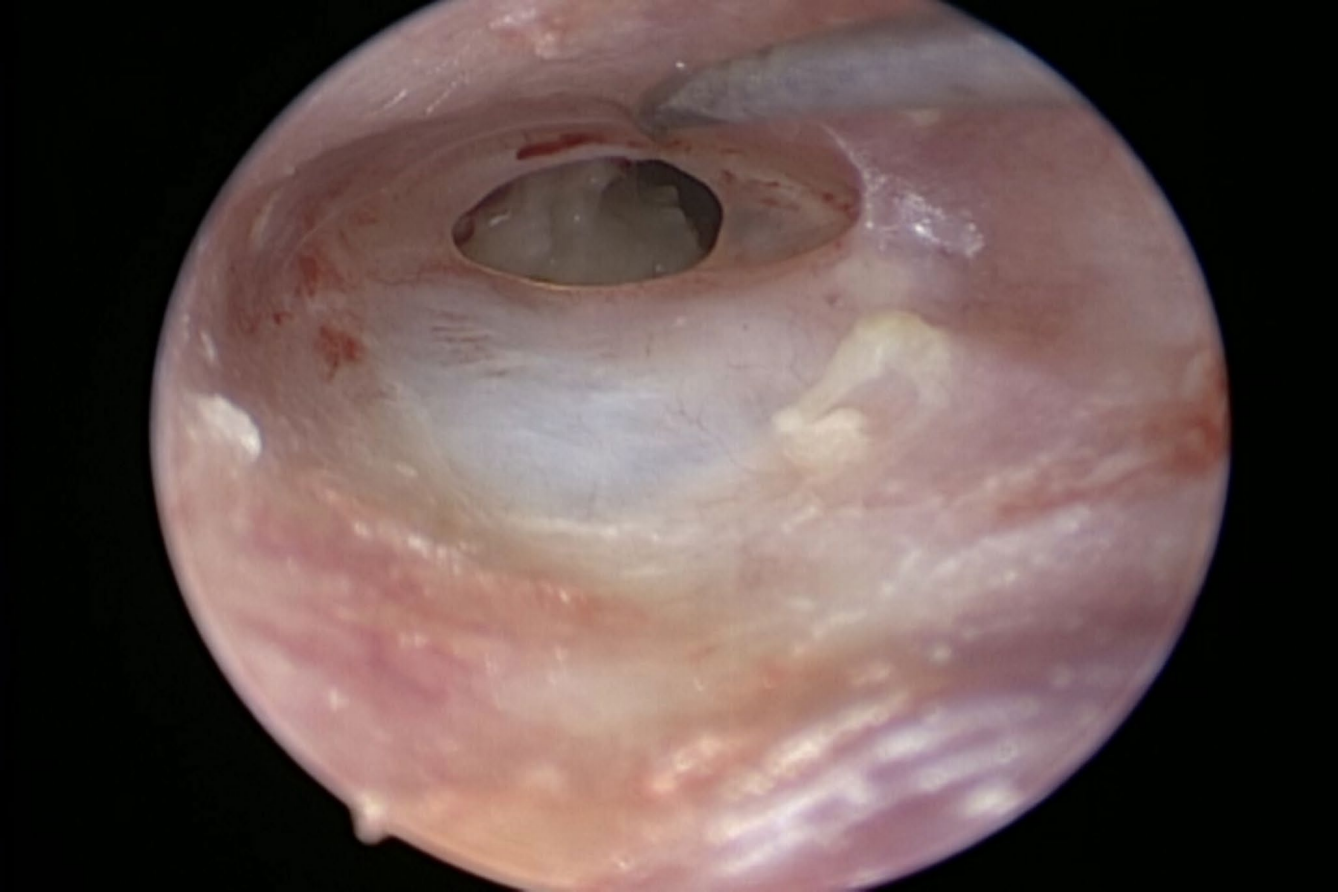
Endoscopic trimming of the anterior edge of the left tympanic membrane perforation with good view despite the anterior canal hump due to the better wide endoscopic view

**Fig. 3:**
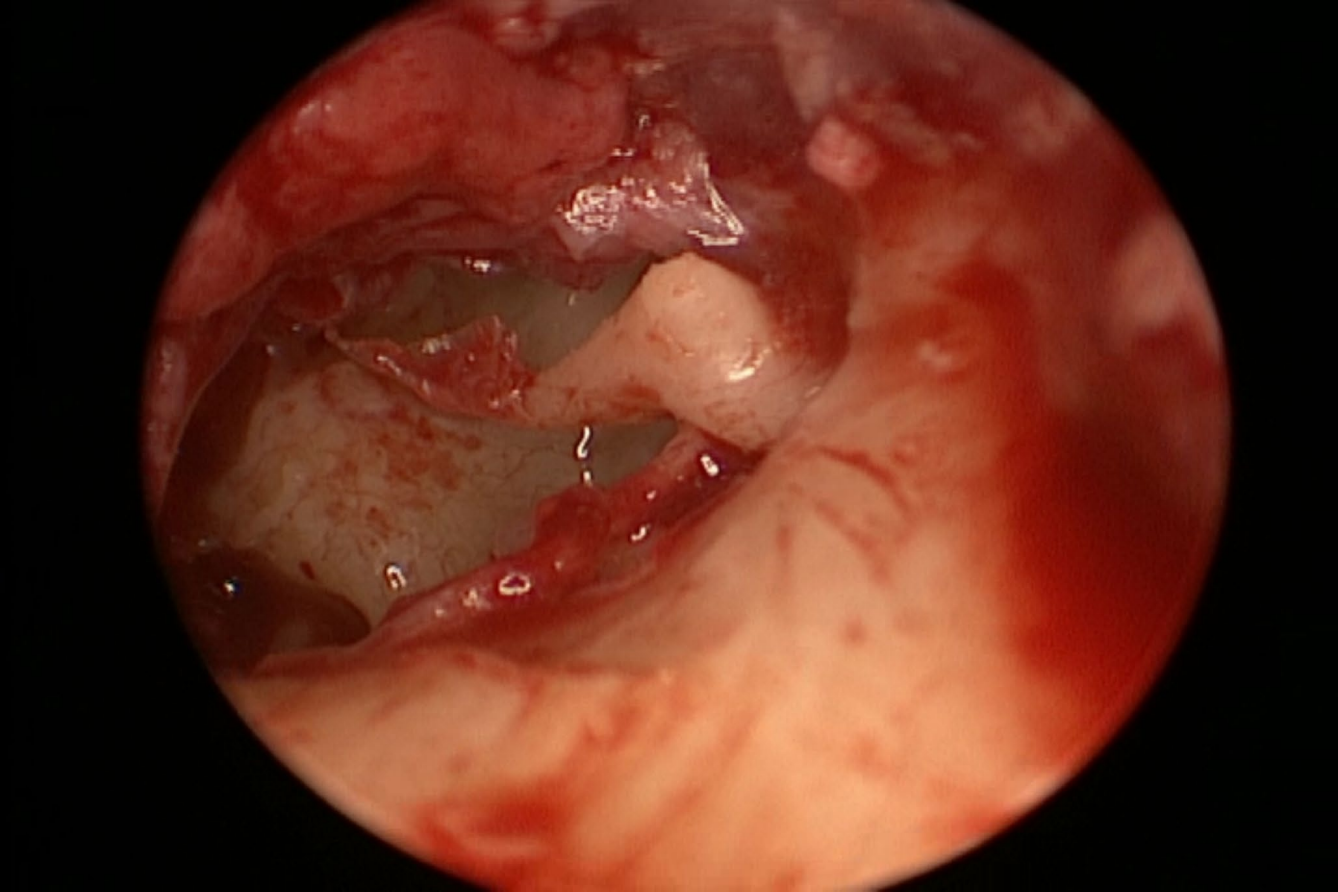
Elevation of the tympanomeatal flap of the left ear with getting of the whole middle ear cavity in view

**Fig. 4:**
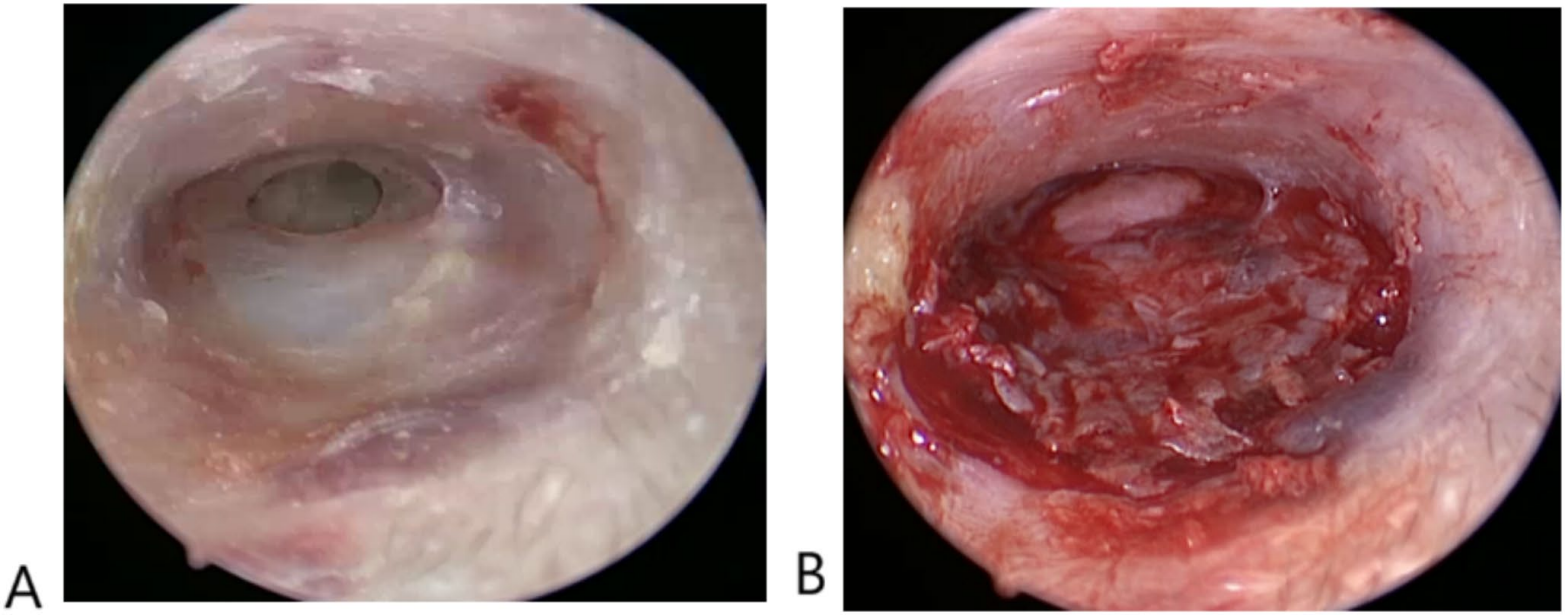
Endoscopic view of the (**A**) left tympanic membrane anterior perforation. (**B**) left tympanic membrane grafting with good view of the whole edges of the perforation

All cases underwent surgery under general anesthesia with a hypotensive modality. 4 mm diameter, 17 cm length Storz zero-degree endoscope was used in the surgery. Storz camera and led light source were attached to the lens and utilized in surgery for visualization and recording. The LED light source was adjusted on 50% illumination (Fig. 1). The surgery started with trimming of the edges of perforation which was performed to refresh the edges to promote healing (Fig. 2). A wide posterior tympanomeatal flap was created from 12 to 6 clock. The flap was elevated and dissected from the malleus and umbo and transposed anteroinferiorly (Fig. 3). Examination of the ossicular chain mobility was performed and ossiculoplasty was performed when needed. Grafting of the TM perforation was performed using temporalis facia in underlay overlay fashion which is placing the graft under the annulus and over the malleus (Fig. 4). Support of the graft was performed by adding layers of absorbable gel foam. The total operative time was recorded starting by the start of endoscopic recording till the end of recording.

All patients were prescribed postoperative antibiotics for 1 week together with nasal decongestants. Local or systemic antibiotics with known ototoxic effect were avoided in the postoperative treatment regimen. The follow up was designed as weekly for the first postoperative month and monthly for the next 6 months. PTA was done three months postoperatively to assess the postoperative hearing state.

Descriptive statistics were calculated using SPSS (Statistical Package for Social Sciences) version 20. Qualitative data were presented as frequency and percentage. Normally distributed quantitative data were presented as mean ± standard deviation (SD). Student’s t-test was used to compare continuous variables. Pearson correlation test was used to assess association correlation.

## Results

From January 2023 to February 2024, 61 cases of chronic suppurative otitis media were identified in the study for endoscopic tympanoplasty. Ten cases were excluded from the study due to missed follow up. The mean age of the included 51 patients was )33.65 ± 10.840) years. The study population included 36 females and 15 males. The operated ears were 30 right ears and 21 left ears. Mean endoscope usage time was (56.57 ± 10.931) minutes. Table 1 summarizes the important audiometric data retrieved from the study including the preoperative and postoperative bone conduction threshold. The current study showed no statistically significant difference between pre and post operative BC (*P* = 0.015). Regarding preoperative mean air bone gap (ABG) it was (20.39 ± 8.114) while the Postoperative mean air bone gap (ABG), it was (7.75 ± 4.039). A statistically significant improvement in ABG was encountered [*P* < 0.001] with better outcome.

**Table 1:** ; Demographic data and comparison between preoperative and postoperative outcome

Surgical group *N* = 51		Test of significance
Age/years (mean ± SD)	(33.65 ± 10.840)	
Mean PreopBC	25.00 ± 6.633	
Mean PostopBC	(25.59 ± 7.046)	*P* = 0.015
Mean PreopABG	(20.39 ± 8.114)	
Mean PostopABG	(7.75 ± 4.039)	*P* < 0.001

In our study we used Pearson correlation test to show the association between duration of endoscopic usage and postoperative BC which showed no statistically significant association between duration of endoscopic usage and postoperative BC (P 0.713).

No postoperative significant vertigo or facial nerve affection were encountered in our cases in the follow up period. In addition, no major complications, such as postoperative bleeding, persistent otorrhea, or profound sensorineural hearing loss, were encountered during the follow-up period.

## Discussion

Endoscopic tympanoplasty is a widely used procedure nowadays. The usage of the endoscope in tympanoplasty enables having a total exposure of TM and the middle ear structures with the ability to look around the corners. In addition, this technique avoids postauricular external incision and extensive soft tissue dissection decreasing the postoperative morbidity and the hospital stay. In addition, EES was engaged in nearly all otological procedures due to the advantages mentioned before. Some drawbacks have been raised as a natural sequence of widespread application of EES. Facial nerve affection and Inner ear problems including vertigo and hearing loss were the raised issues as possible complications of the resultant heat from the endoscope tip while being in the middle ear during surgery [20]. Therefore, this study emphasized the postoperative hearing state, postoperative vertigo and facial nerve outcome in cases of endoscopic tympanoplasty to argue with or against this drawback.

### Bottrill et al. (1996)

reported that the usage of otoendoscopes can cause vertigo due to increasing the temperature around the lateral semicircular canal [21]. Another study was reported by **MacKeith et al. (2008**) that suggested that the tip of rigid endoscopes could cause inner ear tissue damage with the increased temperature [22]. In this study, we did not encounter postoperative significant disabling vertigo in any of our cases.

### Lonsbury-Martin et al. (2014)

suggested that the sensory and neural cells of the cochlea are highly sensitive to environmental factors such as noise, trauma and changes in temperature and outer hair cells are the first cells to be affected which can cause high frequency sensorineural hearing loss [23]. They raised the suspicion of possibility of postoperative high frequency SNHL in cases of EES because of heat induced affection of the outer hair cells of the cochlea. In this study, no statistically significant difference could be identified as regard the bone conduction threshold preoperatively and postoperatively. In addition, we could not identify a statistically significant association between the duration of endoscope usage and the affection of the sensorineural element postoperatively.

### Nomura et al.,

discussed the usage of anti-fog solution to reduce fogging or blurring of the tip of the endoscope due to temperature differences between the endoscopic lens and the warmer surrounding middle ear [24]. Anti-fog solution typically consists of alcohol, surfactants, glycerin and water. They raised the suspicion that anti-fog solution can have a possible ototoxic effect and should be used carefully by applying minimal anti-fog solution to the tip of the endoscope which is then wiped with saline before placement near the middle ear [24]. In our study, we used to put the diluted anti fog solution on a cotton piece and wipe the tip of the lens now and then during surgery. In addition, taking care that the tip of the lens does not touch the middle ear structures is also important. The study showed that neither the generated heat nor the usage of anti-fog solution had a clinically or a statistically significant affection of the bone conduction threshold postoperatively.

Some authors suggested that the facial nerve is more likely to be injured in endoscopic ear surgery than microscopic work because of the intensity of light source that may heat the labyrinthine fluid and nerve structures in the middle ear. This thermogenesis can result in nervous tissue damage, which can manifest as delayed facial nerve paralysis [25, 26]. In our study, we could not identify any case of facial nerve affection during the whole period of the follow up. In addition, the authors studied the role of endoscope in facial nerve decompression in cases of traumatic facial nerve paralysis with promising results [12]. This could prove the concept that the resulting heat from the endoscope does not affect even the whole dehiscent facial nerve.

### Kozin et al. (2014)

performed a study on fresh human temporal bones aimed at measuring temperature changes during rigid middle ear endoscopy in a human temporal bone model and investigate whether suction can act as a significant cooling mechanism [27]. According to their findings, Xenon light sources could cause a temperature rise to 46.9 °C and they recommended the submaximal use of endoscopes and suctioning of the middle ear. Similarly, in this study it is suggested that taking out the endoscope from external auditory canal and cooling it with irrigation plays an important role to avoid thermal damage. **Khan and Parab (2016**) described an endoscope holder-assisted transcanal two-handed endoscopic tympanoplasty procedure with better surgical outcomes. This technique raised the argument regarding the thermal damage of the EES as the light is stable on the middle ear during the whole procedure instead of getting in and out many times during the classic EES. However, they suggested that some simple techniques like irrigation should be used during surgery to prevent thermal damage. In our study, we used the LED light source which generates a lower temperature than the XENON one. In addition, we do not use any kind of endoscope holders as we argue that getting in and out with the endoscope helps decrease the generated temperature and prevents focusing the generated temperature on the middle ear structures.

One limitation of the study is being of retrospective nature and that it focused on endoscopic tympanoplasty only. However, the authors chose to get as many number of cases as we can to help study the effect of endoscopic temperature. in addition, selection of endoscopic tympanoplasty only standardized the disease and helped the study to be more focused on the effect of endoscopic thermogenesis on the inner ear structures. Future studies need to include other endoscopic procedures to argue with or against our conclusion. The authors concluded that the recommended light intensity should not exceed 50% of the light source voltage, along with frequent cooling and misting the tip end with diluted anti-fog solutions. To the best of our knowledge, this is the first study that investigates the effect of heat generated from the light of EES on the sensorineural hearing level based on a series of specific types of EES.

## Conclusion

Endoscopic ear surgery is an expanding field due to the advantages of wide angled view with the ability to look around the corners giving many important details during surgery. Endoscopic tympanoplasty is one of the most common procedures in EES. Some studies raised the suspicion of harmful effects of the thermogenesis generated by the light at the end of the lens on the middle and inner ear structures. In our study, we could not identify any clinically or statistically significant association between the endoscope usage time and the postoperative hearing level. In addition, no facial nerve affection, significant vertigo or postoperative sensorineural hearing loss were noticed after endoscopic tympanoplasty.
